# Is inflammation a missing link between relative handgrip strength with hyperlipidemia? Evidence from a large population-based study

**DOI:** 10.1186/s12944-024-02154-5

**Published:** 2024-05-27

**Authors:** Rui La, Yunfei Yin, Wenquan Ding, Zhiyuan He, Lingchen Lu, Bin Xu, Dinghua Jiang, Lixin Huang, Jian Jiang, Liyu Zhou, Qian Wu

**Affiliations:** 1 Department of Orthopedic Surgery and Sports Medicine, The First Affiliated Hospital of Soochow University, Institute of Orthopedics at Soochow University, Jiangsu, China; 2 Department of Cardiology, The First Affiliated Hospital of Soochow University, Jiangsu, China; 3 Department of Pediatric Surgery and Rehabilitation, Kunshan Maternity and Children’s Health Care Hospital, Jiangsu, China; 4 Department of Cardiology, Shanghai Tenth People’s Hospital, Tongji University School of Medicine, Shanghai, China; 5 Department of Translational Medicine, Seoul National University College of Medicine, Seoul, Korea; 6 Research Institute of Clinical Medicine, Jeonbuk National University Medical School, Jeonju, Korea

**Keywords:** Relative handgrip strength, Hyperlipidemia, National Health and Nutrition Examination Survey, Inflammation, Mediation analysis

## Abstract

**Background:**

Relative handgrip strength (RHGS) was positively correlated with healthy levels of cardiovascular markers and negatively correlated with metabolic disease risk. However, its association with hyperlipidemia remains unknown. The present study investigated the link between RHGS and hyperlipidemia, utilizing data from the National Health and Nutrition Examination Survey (NHANES) and further examined the hypothesis that inflammation may serve a mediating role within this relationship.

**Methods:**

Data were extracted from 4610 participants in the NHANES database spanning 2011–2014 to explore the correlation between RHGS and hyperlipidemia using multivariate logistic regression models. Subgroup analyses were conducted to discern the correlation between RHGS and hyperlipidemia across diverse populations. Additionally, smooth curve fitting and threshold effect analysis were conducted to validate the association between RHGS and hyperlipidemia. Furthermore, the potential mediating effect of inflammation on this association was also explored.

**Results:**

According to the fully adjusted model, RHGS was negatively correlated with hyperlipidemia [odds ratio (OR) = 0.575, 95% confidence interval (CI) = 0.515 to 0.643], which was consistently significant across all populations, notably among women. Smooth curve fitting and threshold effect analysis substantiated the negative association between RHGS and hyperlipidemia. Moreover, the mediating effects analysis indicated the white blood cell (WBC) count, neutrophil (Neu) count, and lymphocyte (Lym) count played roles as the mediators, with mediation ratios of 7.0%, 4.3%, and 5.0%, respectively.

**Conclusions:**

This study identified a prominent negative correlation between RHGS and hyperlipidemia. Elevated RHGS may serve as a protective factor against hyperlipidemia, potentially through mechanisms underlying the modulation of inflammatory processes.

**Supplementary Information:**

The online version contains supplementary material available at 10.1186/s12944-024-02154-5.

## Background

Hyperlipidemia is a metabolic disorder characterized by abnormally elevated lipid components in the blood, particularly cholesterol and triglyceride (TG). Hyperlipidemia poses a considerable threat to human health, including cardiovascular and cerebrovascular incidents, such as coronary heart disease and stroke, as well as digestive disorders, such as fatty liver and pancreatitis [1, 2]. Notably, low-density lipoprotein cholesterol (LDL-C) in the plasma, a pivotal contributor to atherosclerotic disease, was identified as the eighth leading cause of mortality in 2019 worldwide and it accounted for 4.4 million deaths (95% uncertainty interval = 2.35 to 3.76 million), which was 1.4 times greater than that in 1990, according to the Global Burden of Disease Study [3, 4]. However, the high prevalence continues to exert a substantial healthcare burden, despite breakthroughs in therapeutic interventions for hyperlipidemia facilitated by lipid-lowering drugs. This drawback necessitates the early detection of hyperlipidemia and the innovation of preventative measures.

Muscle strength has emerged as a substantial indicator for assessing physical function and forecasting disease risk and has attracted increasing scholarly interest [5]. Handgrip strength (HGS) has been recommended as a reliable measure of muscle strength because of its cost-effectiveness, convenience, and high sensitivity [6, 7]. Studies have demonstrated that decreased HGS was correlated with an increased risk of severe nonalcoholic fatty liver disease, cognitive decline, hospital-associated disability, psychiatric disorders, and osteoporosis [8–12]. However, the physical size of the individual may affect the assessment of health status solely based on HGS [13]. Therefore, researchers have proposed the concept of relative HGS (RHGS), which utilized the body mass index (BMI) to correct the absolute HGS (AHGS), to objectively reflect the combined effects of muscle strength and obesity on health [14, 15]. Elevated RHGS has been positively correlated with healthier levels of cardiovascular markers and negatively correlated with metabolic disease risk [14, 16]. However, RHGS and hyperlipidemia warrant more studies. Nevertheless, it can be tentatively hypothesized that RHGS may also serve as a protective factor against hyperlipidemia.

Inflammation is both a consequence and a trigger of numerous diseases. In a cohort of healthy young adults, a Lifestyle, Biomarkers, and Atherosclerosis study explored the correlation between RHGS and lipid levels, alongside inflammatory markers, and demonstrated that RHGS was negatively correlated with TG (β = -0.15) and high-sensitivity C-reactive protein (CRP) (β = -0.22), suggesting it may affect lipid and inflammation levels [17]. Another cross-sectional study demonstrated a remarkable nonlinear connection between systemic immune-inflammation index and hyperlipidemia [18]. Notably, Ma et al. [19] evaluated systemic inflammation via CRP levels in the context of exploring the nexus between urinary copper and lipids and observed that urinary copper was positively correlated with CRP levels, which in turn was positively correlated with lipid levels, thereby confirming an inflammation-mediated association between copper and lipids. This study also investigated whether inflammation would play a mediating role in the putative negative correlation between RHGS and hyperlipidemia.

Consequently, in light of the predictive implication and protective capacity of RHGS against various health challenges and the intricate interplay among inflammation, muscle strength, and lipid metabolism, the present study aimed to thoroughly investigate the correlation between RHGS and hyperlipidemia through data derived from the National Health and Nutrition Examination Survey (NHANES), a comprehensive health database, and to elucidate the mediating role of inflammation in the association, thus developing new predictors of hyperlipidemia and providing explanations of underlying mechanisms.

## Methods

### Survey description

The NHANES extensively assesses the health and nutritional statuses across the U.S. demographic spectrum, with the approval obtained from the Ethical Review Board of the National Center for Health Statistics. Known for the intricate, multi-stage, stratified, and methodologically rational sampling techniques, alongside the extensive data garnered from a substantial sample size, the NHANES has been pivotal in evaluating population health dynamics and identifying the protective and risk factors associated with diseases over the last decade [20–22]. It has garnered substantial scholarly interest in public health and epidemiological research. Access to the NHANES dataset is facilitated via its online portal, available at no cost to researchers, which also provides detailed elucidation of data collection methodologies.

### Study population

**Fig. 1:**
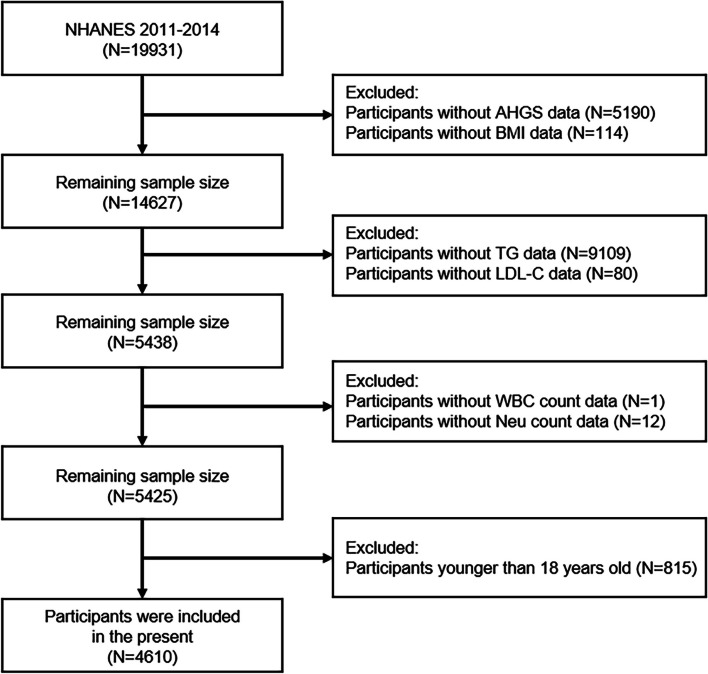
Flowchart of the participant selection from NHANES 2011–2014

Nineteen thousand nine hundred thirty-one participants were enrolled from the two NHANES cycles spanning from 2011 to 2014 in this study. Initially, 5190 and 114 participants were excluded owing to incomplete AHGS and BMI data, respectively, which were essential for the computation of the RHGS. Subsequently, 9109 and 80 participants were excluded because of incomplete TG and LDL-C data, respectively. The remaining participants all had TG, LDL-C, high-density lipoprotein cholesterol (HDL-C), and total cholesterol (TC) data, which were required for the diagnosis of hyperlipidemia. Give that this study also sought to explore the role of inflammation in the association between RHGS and hyperlipidemia, participants without data on inflammatory markers failed to participate in this study, including one lacking white blood cell (WBC) count data and 12 lacking neutrophil (Neu) count data. Additionally, 815 minors were not considered for this study. Ultimately, a total of 4610 participants participated in this cross-sectional study (Fig. 1).

### Assessment of RHGS

The NHAENS provides the protocol and procedure for measuring HGS with an isometric meter. The participants who lost both arms, both hands, or both thumbs or who suffered from paralysis affecting both hands were excluded from the test of HGS. Additionally, the participants that had undergone either wrist or hand surgery within the past 3 months were tested for HGS in the unaffected hand only. The participants were instructed to stand and exert maximal force on the dynamometer with each hand alternately, repeating three times, with a 60-second interlude between successive measurements on the same side to ensure sufficient recovery. AHGS was calculated as an aggregate of the maximal HGS for each hand. Subsequently, RHGS was calculated as the AHGS divided by the BMI, consistent with the previous research [14].

### Assessment of the diagnosis of hyperlipidemia

Consistent with the previous research, in accordance with the Adult Treatment Panel III guidelines of the National Cholesterol Education Program, individuals were diagnosed with hyperlipidemia under one of the following conditions: (1) TG ≥ 150 mg/dL; (2) LDL-C ≥ 130 mg/dL; (3) HDL-C ≤ 40 mg/dL in men and ≤ 50 mg/dL in women; or (4) TC ≥ 200 mg/dL [23]. Furthermore, individuals reporting current use of cholesterol-lowering medications were also considered to have hyperlipidemia [18, 24]. In conclusion, hyperlipidemia was utilized as a dichotomous outcome variable in this study.

### Assessment of inflammatory markers

This present study also investigated the mediating role of inflammatory markers of interest in the relationship between RHGS and hyperlipidemia. Based on previous studies, WBC count, Neu count, and lymphocyte (Lym) count reflect the level of inflammation, and, thus, were included in the mediation analysis as inflammation markers [25, 26]. The NHANES official website explains the laboratory sample processing methods. Briefly, an automatic dilution and mixing device was utilized for sample processing, and Beckman Coulter method of counting and sizing were utilized to measure the complete blood counts. While the Volume, Conductivity and Scatter technology was used to classify the whole blood cells. A Beckman Coulter MAXM was utilized in the 2011–2012 cycle but the equipment changed to Beckman Coulter DXH 800 in the 2013–2014 cycle.

### Covariate definitions

The following variables were considered as the covariates: gender, age, race, education level, household poverty-to-income ratio (PIR), physical activity level, smoking status, drinking status, hypertension status, diabetes status, heart failure status, coronary heart disease status, angina status, heart attack status, stroke status, liver condition, and cancer status. Notably, PIR was categorized into three groups using 1.3 and 3.5 as the thresholds [27]. Similarly, physical activity levels were assessed by the metabolic equivalent (MET) scores. They were categorized into inactive, moderate, and active groups with thresholds of 600 MET-minutes/week and 3000 MET-minutes/week, respectively [28]. Smoking status was classified into never, former, and current groups by inquiring the participants whether they had smoked 100 cigarettes in their lifetime and whether they were currently smoking. Likewise, the participants were assigned to two categories based on whether they had consumed 12 cups of alcohol in their lifetime [29]. Disease condition data were collected through questionnaires; the participants were requested to respond to whether they had been informed about a disease by a doctor or other health professional. Moreover, the participants with missing covariate data were included in the “Unclear” group.

### Statistical analysis

For this cross-sectional study, first, the mean ± standard deviation was used to describe the continuous variables; a Kruskal–Wallis test was conducted to assess the differences. The frequencies and percentages were used to describe the categorical variables, and the chi-square test was conducted to assess the differences. Subsequently, multiple regressions were utilized to examine the correlation between RHGS (the continuous exposure variable) and hyperlipidemia (the binary outcome variable). Three adjusted models were constructed to progressively control for the effects of confounders as follows: an unadjusted crude Model 1; Model 2 adjusted for gender, age, race, education, and PIR; and a sufficiently adjusted Model 3 incorporating all covariates under consideration. Subsequently, a threshold effect analysis and smooth curve fitting were applied to further validate the association between RHGS and hyperlipidemia [30, 31]. Additionally, interaction effect tests were conducted in different subgroups to explore the effects of RHGS on hyperlipidemia in different populations. Finally, statistical mediation effect models centered on three inflammatory markers were developed to explore the role of inflammation in the association between RHGS and hyperlipidemia [32–34]. A two-sided *P*-value < 0.05 was considered to indicate statistical significance. All statistical analyses were conducted via using R software (version 4.2.2) and EmpowerStats (version 4.2).

## Results

### Population baseline characteristics

The detailed baseline characteristics of 4610 participants are presented in Table 1. Within this cohort, 3177 participants were diagnosed with hyperlipidemia, equating to a prevalence of 68.92%. Notably, the prevalence of hyperlipidemia was diminished among men, young adults, Mexican Americans, non-Hispanic Blacks or other races, and participants with higher education levels. Conversely, an elevated prevalence of hyperlipidemia was observed in participants with physical inactivity, along with those who engaged in smoking. Moreover, participants with hypertension, diabetes, heart failure, coronary heart disease, angina, heart attack, stroke, liver condition, or cancer were more likely to suffer from hyperlipidemia. Furthermore, it warrants special attention that participants with hyperlipidemia exhibited prominently higher levels of inflammatory markers and markedly lower AHGS and RHGS than the controls.

**Table 1:** The characteristics of participants

	ALL	Non-Hyperlipidemia	Hyperlipidemia	*P*-value
	*N* = 4610	*N* = 1433	*N* = 3177	
**Gender**				0.004
Male	2282 (49.50%)	755 (52.69%)	1527 (48.06%)	
Female	2328 (50.50%)	678 (47.31%)	1650 (51.94%)	
**Age (year)**				< 0.001
18–44	2166 (46.98%)	965 (67.34%)	1201 (37.80%)	
45–59	1073 (23.28%)	215 (15.00%)	858 (27.01%)	
≥ 60	1371 (29.74%)	253 (17.66%)	1118 (35.19%)	
**Race**				< 0.001
Mexican American	519 (11.26%)	176 (12.28%)	343 (10.80%)	
Other Hispanic	439 (9.52%)	109 (7.61%)	330 (10.39%)	
Non-Hispanic White	1947 (42.23%)	565 (39.43%)	1382 (43.50%)	
Non-Hispanic Black	1006 (21.82%)	347 (24.21%)	659 (20.74%)	
Other Race	699 (15.16%)	236 (16.47%)	463 (14.57%)	
**Education level**				0.010
Below high school	1027 (22.28%)	277 (19.33%)	750 (23.61%)	
High school/GED or equivalent	1015 (22.02%)	316 (22.05%)	699 (22.00%)	
Above high school	2565 (55.64%)	839 (58.55%)	1726 (54.33%)	
Unclear	3 (0.07%)	1 (0.07%)	2 (0.06%)	
**Family PIR**				0.308
≤ 1.29	1491 (32.34%)	490 (34.19%)	1001 (31.51%)	
1.30–3.49	1487 (32.26%)	442 (30.84%)	1045 (32.89%)	
≥ 3.50	1274 (27.64%)	391 (27.29%)	883 (27.79%)	
Unclear	358 (7.77%)	110 (7.68%)	248 (7.81%)	
**Physical activity**				< 0.001
Inactive	1721 (37.33%)	413 (28.82%)	1308 (41.17%)	
Moderate	1485 (32.21%)	495 (34.54%)	990 (31.16%)	
Active	1394 (30.24%)	519 (36.22%)	875 (27.54%)	
Unclear	10 (0.22%)	6 (0.42%)	4 (0.13%)	
**Smoking`**				< 0.001
Never	2599 (56.38%)	862 (60.15%)	1737 (54.67%)	
Former	1002 (21.74%)	263 (18.35%)	739 (23.26%)	
Now	895 (19.41%)	253 (17.66%)	642 (20.21%)	
Unclear	114 (2.47%)	55 (3.84%)	59 (1.86%)	
**Drinking**				0.965
< 12 cups in life	663 (14.38%)	209 (14.58%)	454 (14.29%)	
≥ 12 cups in life	3633 (78.81%)	1127 (78.65%)	2506 (78.88%)	
Unclear	314 (6.81%)	97 (6.77%)	217 (6.83%)	
**Hypertension**				< 0.001
Yes	1599 (34.69%)	291 (20.31%)	1308 (41.17%)	
No	3006 (65.21%)	1140 (79.55%)	1866 (58.73%)	
Unclear	5 (0.11%)	2 (0.14%)	3 (0.09%)	
**Diabetes**				< 0.001
Yes	513 (11.13%)	76 (5.30%)	437 (13.76%)	
No	3982 (86.38%)	1335 (93.16%)	2647 (83.32%)	
Borderline	113 (2.45%)	21 (1.47%)	92 (2.90%)	
Unclear	2 (0.04%)	1 (0.07%)	1 (0.03%)	
**Heart failure**				< 0.001
Yes	148 (3.21%)	22 (1.54%)	126 (3.97%)	
No	4214 (91.41%)	1267 (88.42%)	2947 (92.76%)	
Unclear	248 (5.38%)	144 (10.05%)	104 (3.27%)	
**Coronary heart disease**				< 0.001
Yes	165 (3.58%)	17 (1.19%)	148 (4.66%)	
No	4187 (90.82%)	1269 (88.56%)	2918 (91.85%)	
Unclear	258 (5.60%)	147 (10.26%)	111 (3.49%)	
**Angina**				< 0.001
Yes	102 (2.21%)	16 (1.12%)	86 (2.71%)	
No	4257 (92.34%)	1273 (88.83%)	2984 (93.93%)	
Unclear	251 (5.44%)	144 (10.05%)	107 (3.37%)	
**Heart attack**				< 0.001
Yes	170 (3.69%)	16 (1.12%)	154 (4.85%)	
No	4191 (90.91%)	1273 (88.83%)	2918 (91.85%)	
Unclear	249 (5.40%)	144 (10.05%)	105 (3.31%)	
**Stroke**				< 0.001
Yes	152 (3.30%)	23 (1.61%)	129 (4.06%)	
No	4209 (91.30%)	1265 (88.28%)	2944 (92.67%)	
Unclear	249 (5.40%)	145 (10.12%)	104 (3.27%)	
**Liver condition**				< 0.001
Yes	175 (3.80%)	40 (2.79%)	135 (4.25%)	
No	4184 (90.76%)	1248 (87.09%)	2936 (92.41%)	
Unclear	251 (5.44%)	145 (10.12%)	106 (3.34%)	
**Cancer**				< 0.001
Yes	385 (8.35%)	87 (6.07%)	298 (9.38%)	
No	3978 (86.29%)	1202 (83.88%)	2776 (87.38%)	
Unclear	247 (5.36%)	144 (10.05%)	103 (3.24%)	
**WBC count (1000 cells/uL)**	6.78 ± 2.24	6.45 ± 1.97	6.92 ± 2.34	< 0.001
**Neu count (1000 cells/uL)**	3.96 ± 1.67	3.73 ± 1.59	4.06 ± 1.69	< 0.001
**Lym count (1000 cells/uL)**	2.04 ± 1.03	1.98 ± 0.63	2.07 ± 1.17	0.002
**AHGS (kg)**	70.53 ± 21.76	73.03 ± 21.51	69.41 ± 21.79	< 0.001
**BMI (kg/m** ^**2**^ **)**	28.66 ± 6.97	26.21 ± 5.99	29.77 ± 7.10	< 0.001
RHGS (kg/kg*m ^**−2**^ **)**	2.57 ± 0.92	2.89 ± 0.95	2.43 ± 0.87	< 0.001
**TG (mg/dL)**	113.24 ± 65.03	73.97 ± 28.81	130.95 ± 68.95	< 0.001
**LDL-C (mg/dL)**	111.72 ± 35.39	92.47 ± 19.86	120.40 ± 37.38	< 0.001
**HDL-C (mg/dL)**	53.88 ± 15.48	58.79 ± 11.77	51.67 ± 16.42	< 0.001
**TC (mg/dL)**	188.25 ± 40.64	166.05 ± 21.49	198.26 ± 43.19	< 0.001

### Association between RHGS and hyperlipidemia

The association between RHGS and hyperlipidemia was delineated in Table 2. All three models indicated that continuous RHGS was negatively correlated with hyperlipidemia, particularly in the well-adjusted Model 3, where the risk of hyperlipidemia decreased by 42.5% for each unit augmentation in RHGS [odds ratio (OR) = 0.575, 95% confidence interval (CI) = 0.515 to 0.643]. Moreover, RHGS was segmented into quartiles to scrutinize the inverse correlation between RHGS and hyperlipidemia. The results from all models uniformly indicated that the association between elevated RHGS and a diminished risk of hyperlipidemia was statistically significant, and this relationship persisted across groups (*P* for trend < 0.001). Notably, in the group Q4 after adjusting for all covariates, the risk of hyperlipidemia was 31.8% of the previous risk with an increase of each RHGS unit. In addition, the multiple regression results for the association between RHGS and the four kinds of serum lipids (continuous variables) were shown in the Supplementary Table 1.

**Table 2:** The relationship between RHGS and hyperlipidemia

	OR (95% CI), *P*-value		
	Model 1^a^	Model 2^b^	Model 3^c^
	Hyperlipidemia		
**RHGS (continuous)**	0.582 (0.543, 0.624), < 0.001	0.534 (0.480, 0.593), < 0.001	0.575 (0.515, 0.643), < 0.001
**Q1 (0.502–1.884)**	1.0 (Reference)	1.0 (Reference)	1.0 (Reference)
**Q2 (1.885–2.444)**	0.500 (0.409, 0.612), < 0.001	0.548 (0.443, 0.678), < 0.001	0.604 (0.485, 0.751), < 0.001
**Q3 (2.444–3.176)**	0.333 (0.273, 0.405), < 0.001	0.317 (0.251, 0.399), < 0.001	0.370 (0.291, 0.469), < 0.001
**Q4 (3.176–7.019)**	0.274 (0.226, 0.333), < 0.001	0.256 (0.196, 0.336), < 0.001	0.318 (0.239, 0.422), < 0.001
***P *** **for trend**	0.579 (0.535, 0.627), < 0.001	0.550 (0.488, 0.620), < 0.001	0.606 (0.535, 0.687), < 0.001

^a^Model 1: No covariates were adjusted

^b^Model 2: Adjusted for gender, age, race, education level, family PIR

^c^Model 3: Adjusted for gender, age, race, education level, family PIR, physical activity, smoking status, drinking status, hypertension status, diabetes status, heart failure status, coronary heart disease status, angina status, heart attack status, stroke status, liver condition, cancer status

### Subgroup analyses

**Fig. 2:**
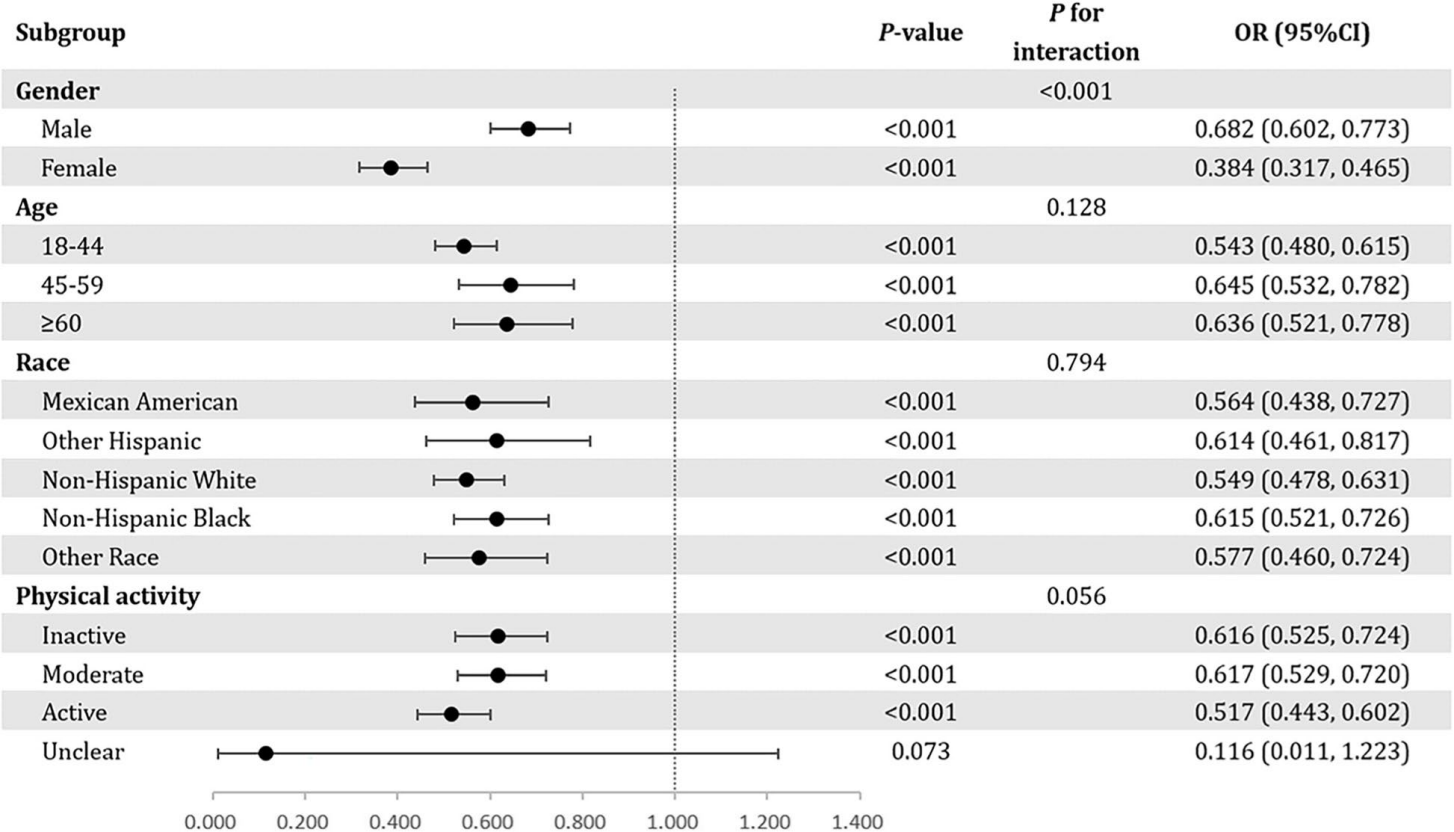
Forest plot of subgroup analyses for the associations between RHGS and hyperlipidemia: adjusted for gender, age, race, education level, family PIR, physical activity, smoking status, drinking status, hypertension status, diabetes status, heart failure status, coronary heart disease status, angina status, heart attack status, stroke status, liver condition, and cancer status

Subgroup analyses were conducted to elucidate the connection between RHGS and hyperlipidemia in different populations (Fig. 2). Overall, the negative associations between RHGS and hyperlipidemia were all statistically significant across various subgroups. Age, race, and physical activity had no interaction effect on the association between RHGS and hyperlipidemia, whereas sex exerted a modifying effect (*P* for interaction < 0.001). Moreover, the inverse correlation between elevated RHGS and decreased risk of hyperlipidemia was markedly pronounced in women (OR = 0.384, 95% CI = 0.317 to 0.465).

### Smooth curve fitting and threshold effect analysis

**Fig. 3:**
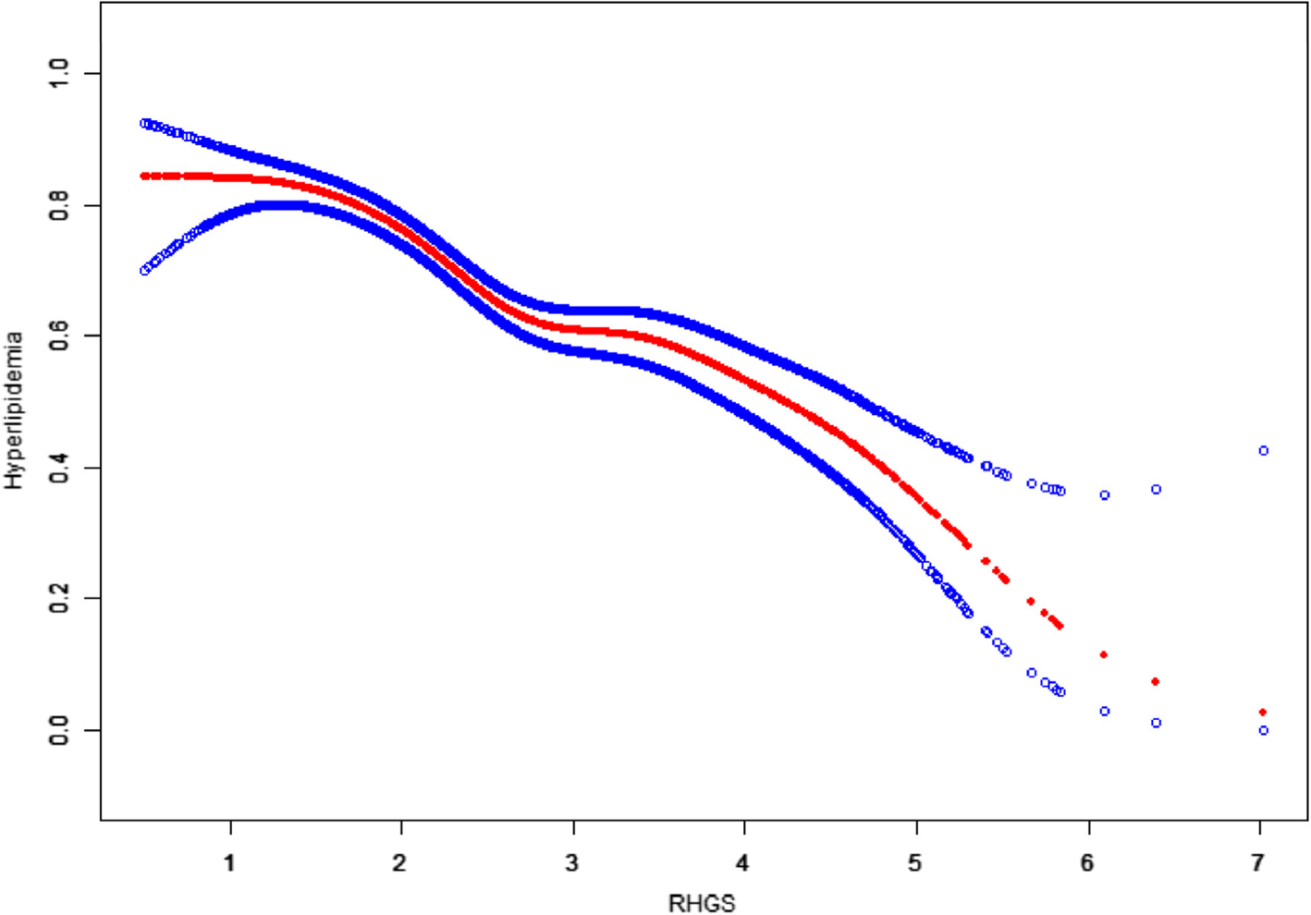
Smooth curve fitting for RHGS and hyperlipidemia: adjusted for gender, age, race, education level, family PIR, physical activity, smoking status, drinking status, hypertension status, diabetes status, heart failure status, coronary heart disease status, angina status, heart attack status, stroke status, liver condition, and cancer status. The red line in the center represents the OR and the blue lines on either side of it represent the 95% CI.

The smooth curve fitting model demonstrated a negative correlation between RHGS and hyperlipidemia (Fig. 3). Moreover, the threshold effect analysis also suggested an inverse correlation between RHGS and hyperlipidemia, despite there was an inflection point of 2.611, both before and after which RHGS was negatively associated with hyperlipidemia as well (Table 3).

**Table 3:** Threshold effect analysis of RHGS and hyperlipidemia

Hyperlipidemia	Adjusted OR (95% CI), P -value
RHGS	
Linear regression model	0.575 (0.515, 0.643), < 0.001
Two-segment piecewise linear regression model	
Inflection point	2.611
RHGS < Inflection point	0.451 (0.363, 0.560), < 0.001
RHGS > Inflection point	0.652 (0.565, 0.754), < 0.001
Log-likelihood ratio	0.009

Adjusted for gender, age, race, education level, family PIR, physical activity, smoking status, drinking status, hypertension status, diabetes status, heart failure status, coronary heart disease status, angina status, heart attack status, stroke status, liver condition, cancer status.

### Mediation analysis

**Fig. 4:**
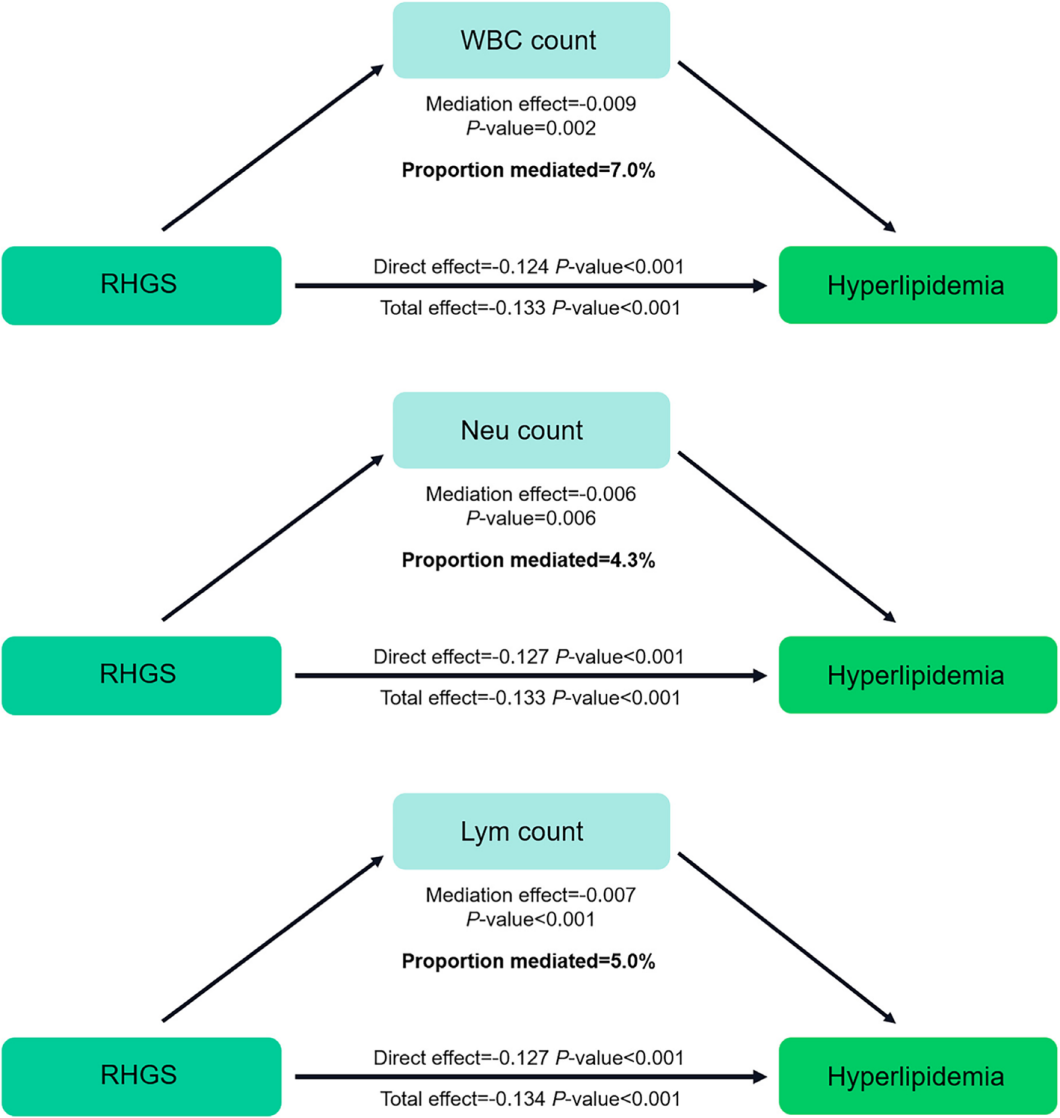
Mediation effect of inflammatory markers for the association between RHGS and Hyperlipidemia: adjusted for gender, age, race, education level, family PIR, physical activity, smoking status, drinking status, hypertension status, diabetes status, heart failure status, coronary heart disease status, angina status, heart attack status, stroke status, liver condition, and cancer status

The present study further investigated the role of inflammatory markers in the association between RHGS and hyperlipidemia via a mediation analysis. The results suggested that the WBC, Neu, and Lym counts played remarkable mediating roles (*P*-values for mediation effects were 0.002, 0.006, and < 0.001, respectively), with mediating ratios of 7.0%, 4.3%, and 5.0%, respectively (Fig. 4).

## Discussion

The present study enrolled 4610 participants from the NHANES spanning from 2011 to 2014 to investigate the correlation between RHGS and hyperlipidemia through a cross-sectional study. A total of 3177 participants were diagnosed with hyperlipidemia, suggesting a prevalence of 68.92% within the cohort, similar to previous findings [35]. Multiple logistic regression analysis, smooth curve fitting, and threshold effect analysis indicated and further confirmed the inverse correlation between RHGS and hyperlipidemia, with the subgroup analyses suggesting a continuity of the association. Ultimately, the mediation analysis indicated inflammation as one of the mechanisms underlying the association between RHGS and hyperlipidemia. In summary, this study demonstrated the great potential of enhancing muscle strength in affecting hyperlipidemia, which deserves more investigation.

The high prevalence and risk of hyperlipidemia warrant additional research for predictive and preventive methods to minimize the threat to cardiovascular and cerebrovascular health. Several epidemiological studies have demonstrated the risk factors for hyperlipidemia by incorporating metallic elements, snoring, and depression, and protective factors, such as a healthy lifestyle, including, the supplementation of folate, fruits, and vegetables as well as physical activity, weight control, and reduced alcohol consumption [24, 36–42]. Particularly, RHGS can be enhanced by physical activity and weight control. It appears to be an effective tool for predicting and improving hyperlipidemia.

RHGS is emerging as a research hotspot, particularly in disease prediction. First, a prospective cohort study based on the UK Biobank suggested that RHGS was negatively associated with endometrial, liver, gallbladder, kidney, esophageal, pancreatic, colorectal, breast, and all-cause cancers; furthermore, it outperformed AHGS in predicting gastric cancer [43]. Moreover, a cross-sectional study demonstrated that RHGS was negatively associated with the Patient Health Questionnaire-9 scores used to assess depression, in the adjusted model for Korean adults (β for right RHGS = -0.76; β for left RHGS = -0.83) [44]. Another prospective study also validated that the RHGS can predict the risk of depression among middle-aged and elderly individuals in China [45]. Furthermore, high RHGS also plays a prominent role in coping with chronic health problems. Reduced RHGS levels increase the risk of prediabetes in men [46]. Moreover, elevated RHGS levels decrease the risk of diabetes in women [47]. More importantly, researchers have recognized the great significance of enhancing RHGS in maintaining cardiovascular safety. Improved RHGS has been correlated with a reduced risk of hypertension in Chinese as well as Korean populations, while RHGS diminishes with age, suggesting the need for improved muscle strength in prevention and treatment of hypertension in the aging population [48, 49]. Existing studies have also comprehensively reviewed the relationship between RHGS and cardiovascular health markers; RHGS was positively associated with beneficial HDL-C and apolipoprotein (Apo) A1 and negatively associated with harmful waist circumference, body fat percentage, TG, and Apo B [17]. These results strongly support the importance of increasing RHGS in reducing the risk of cardiometabolic disorders. Enlightened by the above studies, this work probed the association between RHGS and hyperlipidemia, and indicated similar protective effects originating from RHGS, which are applicable in different populations, particularly in women. Moreover, these findings paralleled those of a previous study that suggested that RHGS, despite being lower in women than in men, improved lipid levels more remarkably, indicating a greater benefit of RHGS in limiting the risk of hyperlipidemia in women [14]. This phenomenon may be attributed to the role of increasing muscle strength, such as exercise training, in promoting the expression of aromatase, a key enzyme in estrogen synthesis, in skeletal muscles as well as in increasing estrogen which can improve lipid metabolism in women [50, 51]. Thus, the protective effect of muscle strength against hyperlipidemia was amplified in women.

Despite the present study confirmed a marked negative association between RHGS and hyperlipidemia, the reasons underlying this phenomenon remain unclear. On the one hand, it needs to be emphasized that the present study confirmed the mediating effect of inflammation in the link between RHGS and hyperlipidemia, which was a bright spot in explaining the mechanism involved. Previous researches have examined the relationship between muscle status and inflammation. Tan et al. [52] calculated the skeletal muscle mass index, which was negatively associated with interleukin-6 (IL-6) levels. Besides, another study with a similar design to the current study, explored the relationship between RHGS and nonalcoholic fatty liver disease and the mediating role of inflammation therein. It demonstrated a negative correlation between RHGS and CRP, and nonalcoholic fatty liver disease [53]. Volaklis et al. [54] investigated the association between muscle strength and inflammatory markers in patients with heart disease and obtained similar results, where low muscle strength was correlated with increased CRP. These findings highlight the anti-inflammatory properties of high RHGS. In turn, inflammation affects the development of hyperlipidemia as well [18]. Moreover, patients with both inflammatory bowel disease and spondyloarthritis suffer from dyslipidemia, suggesting the impact of long-term chronic inflammation on lipid levels [55, 56]. Therefore, it is reasonable to hypothesize that inflammation is a bridge between RHGS and hyperlipidemia. It would make sense to adjust RHGS to modify the level of inflammation to reduce the risk of hyperlipidemia. On the other hand, skeletal muscles are not only motor organs but also endocrine organs that secrete myocytokines. A high level of RHGS also indicates that the muscle remains in a healthy state and can function well, secreting adequate amounts of beneficial myocytokines. IL-15 is a typical representative which both acts on the skeletal muscle and maintaining its metabolic homeostasis by reducing muscle protein degradation, and reduces lipid deposition in adipocytes to reduce fat mass [57]. Irisin is another well-known myocytokine modulating lipid levels by increasing adipocyte energy expenditure to reduce lipid accumulation while regulating oxidative metabolism in the myocytes [57]. In addition, myocytokines are central to ameliorating insulin resistance, which is one of the indirect pathways involved in regulating lipid metabolism. Myocytokines augment insulin sensitivity, which increases the uptake and utilization of glucose and prevents its excessive conversion to lipids, thus maintaining stable lipid levels [58]. In summary, complex mechanisms govern the association between RHGS and hyperlipidemia and involve multiple aspects, such as inflammation and endocrinology, thus warranting additional detailed studies in the future.

## Strengths and limitations

Encouragingly, the present study first explored the relationship between RHGS and hyperlipidemia. It demonstrated a negative correlation between RHGS and hyperlipidemia, thus indicating high RHGS as a potential protective factor against hyperlipidemia. Moreover, this study utilized a mediation analysis to investigate the role of inflammation in the relationship between RHGS and hyperlipidemia, partly explaining the mechanism underlying this association. Furthermore, the present study offered insights into the future prevention of hyperlipidemia by improving muscle strength.

However, the current study also contains some limitations. First, the present study is cross-sectional, proposing the hypothesis that RHGS may affect hyperlipidemia partly by changing the inflammatory levels and evaluating the plausibility of this hypothesis. Nonetheless, it still fails to obtain a clear causality, thus warranting confirmation by future prospective and experimental studies. In addition, there are still many factors not included in the consideration of covariates, which may have affected the results. Furthermore, this study utilized data from only the U.S. population, which affected the reliability of the results. Future inclusion of other races is strongly required to improve the applicability of the findings.

## Conclusion

In conclusion, the present study confirmed the strong association between elevated RHGS and reduced risk of hyperlipidemia, and explained that the one of the potential mechanism of linking RHGS and hyperlipidemia was the mediation of inflammation, which broadens the horizons for the prevention of hyperlipidemia. The enhancement of muscle strength to modulate inflammation may be an effective measure to prevent hyperlipidemia in the future. More researches are required to further elaborate the latent mechanisms and validate the causal associations to compensate for the limitations of this cross-sectional study.

### Supplementary Information

Supplementary Material 1.

## Data Availability

The data underlying this article are available in NHANES, at https://wwwn.cdc.gov/Nchs/Nhanes/.

## Abbreviations

AHGS: absolute HGS
Apo: apolipoprotein
BMI: body mass index
CI: confidence interval
HDL-C: high-density lipoprotein cholesterol
HGS: handgrip strength
IL: interleukin
LDL-C: low-density lipoprotein cholesterol
Lym: lymphocyte
MET: metabolic equivalent
Neu: neutrophil
NHANES: National Health and Nutrition Examination Survey
OR: odds ratio
PIR: poverty-to-income ratio
RHGS: relative handgrip strength
TC: total cholesterol
TG: triglyceride
WBC: white blood cell
