# Supplementary material for: Is inflammation a missing link between relative handgrip strength with hyperlipidemia? Evidence from a large population-based study
Source: Lipids Health Dis. 2024 May 27;23:159. doi: 10.1186/s12944-024-02154-5 (PMC11131302; doi:10.1186/s12944-024-02154-5)
Supplement: Supplementary file 1 — Supplementary Material 1. [file 12944_2024_2154_MOESM1_ESM.docx]

| Supplementary Table 1: The relationship between RHGS and serum lipids. | | | |  |  |
| --- | --- | --- | --- | --- | --- |
|  | **OR (95% CI), *P*-value** | | | | |
|  | **TG (mg/dL)** | **LDL-C (mg/dL)** | **HDL-C (mg/dL)** | **TC (mg/dL)** | |
| **RHGS (continuous)** | -13.354 (-16.257, -10.450), <0.001 | -3.226 (-4.844, -1.608), <0.001 | 4.815 (4.156, 5.474), <0.001 | -1.091 (-2.928, 0.747), 0.245 | |
| **Q1 (0.502-1.884)** | 1.0 | 1.0 | 1.0 | 1.0 | |
| **Q2 (1.885-2.444)** | -14.857 (-20.182, -9.533), <0.001 | -4.506 (-7.468, -1.543), 0.003 | 4.850 (3.639, 6.061), <0.001 | -2.642 (-6.006, 0.722), 0.124 | |
| **Q3 (2.444-3.176)** | -23.041 (-29.110, -16.972), <0.001 | -7.217 (-10.594, -3.841), <0.001 | 7.853 (6.472, 9.233), <0.001 | -3.992 (-7.827, -0.158), 0.041 | |
| **Q4 (3.176-7.019)** | -29.609 (-36.918, -22.299), <0.001 | -6.107 (-10.174, -2.041), 0.003 | 10.989 (9.326, 12.651), <0.001 | -1.061 (-5.679, 3.558), 0.653 | |
| ***P* for trend** | -13.000 (-16.292, -9.708), <0.001 | -2.612 (-4.443, -0.780), 0.005 | 4.881 (4.132, 5.630), <0.001 | -0.339 (-2.418, 1.740), 0.749 | |
| Adjusted for gender, age, race, education level, family PIR, physical activity, smoking status, drinking status, hypertension status, diabetes status, heart failure status, coronary heart disease status, angina status, heart attack status, stroke status, liver condition, cancer status. | | | | | |
|  | | | | |  |
